# The Arrival of the Metaverse in Neurorehabilitation: Fact, Fake or Vision?

**DOI:** 10.3390/biomedicines10102602

**Published:** 2022-10-17

**Authors:** Rocco Salvatore Calabrò, Antonio Cerasa, Irene Ciancarelli, Loris Pignolo, Paolo Tonin, Marco Iosa, Giovanni Morone

**Affiliations:** 1 IRCCS Centro Neurolesi “Bonino-Pulejo”, 98123 Messina, Italy; 2 Institute for Biomedical Research and Innovation (IRIB), National Research Council of Italy, 98164 Messina, Italy; 3 Pharmacotechnology Documentation and Transfer Unit, Preclinical and Translational Pharmacology, Department of Pharmacy, Health Science and Nutrition, University of Calabria, 87036 Calabria, Italy; 4 S. Anna Institute, 1680067 Crotone, Italy; 5 Department of Life, Health and Environmental Sciences, University of L’Aquila, 67100 L’Aquila, Italy; 6 Department of Psychology, Sapienza University of Rome, 00185 Rome, Italy; 7 Santa Lucia Foundation IRCSS, 00179 Roma, Italy; 8 San Raffaele Institute of Sulmona, 67039 Sulmona, Italy

**Keywords:** metaverse, neurorehabilitation, virtual reality, artificial intelligence, body schema representation, multisensory feedback, movement disorders, cognitive impairment

## Abstract

The metaverse is a new technology thought to provide a deeper, persistent, immersive 3D experience combining multiple different virtual approaches in a full continuum of physical–digital interaction spaces. Different from virtual reality (VR) and augmented reality (AR), the metaverse has a service-oriented solid model with an emphasis on social and content dimensions. It has widely been demonstrated that motor or cognitive deficits can be more effectively treated using VR/AR tools, but there are several issues that limit the real potential of immersive technologies applied to neurological patients. In this scoping review, we propose future research directions for applying technologies extracted from the metaverse in clinical neurorehabilitation. The multisensorial properties of the metaverse will boost the embodied cognition experience, thus influencing the internal body representations as well as learning strategies. Moreover, the immersive social environment shared with other patients will contribute to recovering social and psychoemotional abilities. In addition to the many potential pros, we will also discuss the cons, providing readers with the available information to better understand the complexity and limitations of the metaverse, which could be considered the future of neurorehabilitation.

## 1. Introduction

The metaverse is a conceptualization of the next—virtual, hypercomplex, digital—internet, thought to provide new opportunities for human communication while blurring avatars of themselves [1]. The main feature of the metaverse should be the fusion between the virtual world and the physical one [2,3]. Born as an add-on feature of gaming companies, the metaverse has been proposed as the key to speeding up the shift from the actual economy to a digital economy. Despite this might sound like a technological fantasy, famous global companies, investors, policy makers and politicians have been attracted by the cultural, social and economic opportunities that the metaverse will bring to us. 

The core of the metaverse is the extended reality (XR) technologies, which include virtual reality (VR), augmented reality (AR) and mixed reality. The metaverse should work in the same way as the actual VR application to simulate a hybrid experience that people can manipulate and explore as if they were there. There are important differences between VR/AR applications and the XR [4]. First, VR and AR are essentially simulation technologies that are designed to replicate (VR) the real world with a virtual one, whereas AR supplements the real world with virtual content by overlaying digital images on physical objects. Otherwise, the metaverse has a strong service-oriented model with an emphasis on social and content dimensions. XR technologies are already used in entertainment, tourism and telemedicine (tele-surgery) industries for integrating digital information layers into physical spaces (through smartphones, tablets, etc.) and bridging them with the digital ones. XR devices produce realistic and highly immersive experiences by artificially reconstructing various human senses through the “federation” of multiple technologies (e.g., tangible interfaces, artificial intelligence, Internet of Things). The main innovation of XR with respect to VR/AR tools is related to the haptic technologies, which enable seamless interaction between the real and the virtual world. The new haptic technologies embedded in the metaverse platform should replicate various tactile sensations generated by interactions with the real environment (i.e, dynamic variations in pressure, shear forces and temperature, with sensors and actuators) into the virtual world.

Despite the debate about the nature and definition of the metaverse, this does not preclude further application of this technology in medicine, which could extend the scenarios and possibilities already exploited with VR and AR. In the last twenty years, a plethora of meta-analytic and systematic reviews have provided sufficient information to support evidence-based care practice guidelines for treating behavioral, motor, cognitive and emotional symptoms using VR therapy. However, the degree of this effectiveness has not been well defined yet. The limited levels of immersion and interaction provided by the actual wearable electronic devices, the moderate effect size of VR-related therapies with respect to traditional treatments and the lack of a cost-benefit analysis might influence the real power of this technological domain in clinical neurorehabilitation. For this reason, the most important challenge for the future is the development of new clinical protocols based on innovative technology capable of bridging the gap between the virtual space and the physical space by exploiting shared computational scenarios, haptic technologies and homogenous therapeutic approaches. Indeed, as established by the GBD (Global Burden of Diseases, Injuries, and Risk Factors Study [5]), in the last thirty years, neurological disorders are recognised as the main cause of disability and the second leading cause of death worldwide, with rates of 15% and 39%, respectively, for the incidence of disability and death. Thus, the development of new clinical protocols aimed at reducing this impact is mandatory.

Starting from these premises, this paper discusses the potential of the metaverse in neurorehabilitation by bringing together the more recent technological advancements with the recent studies from clinical neurology related to empowerment induced by immersive VR experiences. Considering that the metaverse cannot already be applied to medical purposes because of considerable technical hurdles that need to be solved, the theoretical background to the application of this technology in neurological disorders leads to the integration of multiple technologies for facilitating the virtuality–reality interconnection between patients. In particular, the creation of a new multi-sensory and multi-user experience of the physical presence designed for enhancing the senses of presence, body ownership and agency could help clinicians in boosting neural plasticity and functional recovery in critical brain regions involved in the pathophysiological mechanisms of specific neurological disorders. With this in mind, and also considering the neurological disorders responding better to VR therapies, we hypothesize that people affected by motor disorders (i.e., stroke, multiple sclerosis) and cognitive (i.e., hemispatial neglect, memory) and emotional (i.e., pain) impairments will greatly benefit from the translation of this new technology in clinical protocols. In this perspective paper, we also explored the differences between existing VR/AR tools, identifying the possible pitfalls related to the use of the “metaverse” term.

## 2. Metaverse in Medicine

The entry of the metaverse into a medical context was proposed by Yang et al. [6] and Wiederhold and Riva [3]. 

Yang and colleagues [6] conceptualized this kind of platform as a new tool for integrating medical doctors and patients in a virtual space where the entire clinical care process (diagnosis, examination, evaluation of treatment diseases, in-home care, consultation) would be realized by means of internet access to a 3D computer-generated social environment. This would be challenging given the rising number of patients requiring online mental health services. Similarly, Mescko [7] hypothesized that the metaverse, in the cardiovascular domain, would replace online patient forums or current telemedical visits, extending until to becoming a virtual university where lecturers could teach students the inner workings of the cardiovascular system in 3D. The translation of metaverse platforms in medicine could replace the actual advanced technological tools, such as telehealth systems, smartphones and chatbots. 

Extending this vision, Wiederhold and Riva [3] suggested that the metaverse can be seen as the convergence of three current key technical trends: embodiment and presence, digital twinning, and blockchain. These three features could be used to provide whole new methods of providing treatment, potentially reducing costs and significantly raising patient outcomes.

Furthermore, digital medicine had fast development during the SARS-CoV2 pandemic and related lockdowns. In particular, physicians and patients recognized the utility of telemedicine as a valid clinical monitoring tool that also allowed for psychometric assessment and behavioral screening [8].

The metaverse technologies, combining VR and internet connection, will be an outstanding innovation in the future of immersive experiences. Indeed, starting from the two-dimensional display of a traditional computer around the 1980s, the communication between humans and technology in a medical context developed with the introduction of VR systems around the early 1990s [9,10]. Immersive VR systems exploit realistic 3D graphics, stereoscopic viewing and head tracking to create interactive, first-person experiences that can be more ecologically valid than traditional, noninteractive experimental stimuli and produce user physiological responses that mimic real-world experiences [11]. Up to now, VR has been used clinically to make people believe that something that is not present is real. However, the hybrid dimension of the metaverse can also fool the predictive coding mechanisms that regulate our bodily experience, making people feel “real” in situations that are not. This is achieved through the integration of different embodied technologies (e.g., haptic and interoceptive technologies), by increasing the transposition of users’ viewpoint in a first-/third-person perspective, and by taking advantage of big data analysis from AI algorithms to enhance immersive experience and enable human-like intelligence of virtual agents [12].

In conclusion, the fundamental innovations that could be brought about by the metaverse rely on the evolution of immersive experience and on the employment of multiple technologies—artificial intelligence, internet of things, blockchain, etc.—to facilitate the virtuality–reality interconnection by mimicking brain functioning. In particular, AI tools (i.e., deep learning) [13] will bring insights into new treatment approaches, leading to a profound impact on personalized medicine for mental health conditions.

## 3. VR in Neurorehabilitation: The State-of-the-Art 

First of all, it is important to note a common improper use of the term “virtual reality” in clinical settings, especially in neurorehabilitation. VR is more than a simple display of virtual images: It can bring the observer inside the virtual environment surrounding him/her using a 3D computer-generated representation, allowing the subject to move around this virtual world, to see it from different angles and to interact with the virtual objects responding in real time to the movements of the body in a naturalistic way [14]. 

The number and range of the user’s sensory and motor channels connected to the system determine the immersivity. The possibility of interaction with the virtual world and the exploitation of the subject’s imagination influence: (a) the sense of presence in the virtual environment, (b) the sense of body ownership related to the overlapping or real body with the avatar’s body and (c) the sense of agency [14,15].

There are mainly three different technological solutions providing the patients with the abovementioned features of VR:

- The most common and probably the most used in the future for its reduced costs and easiness to use are the head-mounted displays (HMD). HMDs are wearable devices composed of two small displays mounted close to the eyes, with a head-tracking system that updates the binocular images according to the observer’s head movements, with earphones to deliver audio stimulation, and the most recent devices also include a system for tracking hand movements [14].
- A secondary solution is the powerwall screen composed of a large high-resolution back projected screen of 3D glass combined with an optical or ultrasonic tracking system for recording (and translating into the virtual world in real-time) the positions of the head and hands. This system is partially immersive, and probably it will not be used in metaverse applications.
- The last solution is very expensive (and it could limit its diffusion) and is the *CAVE*, acronym for cave automatic virtual environment. This usually consists of a square room composed of four or six (if floor and ceiling are included) back-projected screens joined together (forming the walls of the room) that, combined with the dedicated glass for 3D vision, provide a continuous projection surface and head- and hand-tracking devices.

In neurorehabilitative clinical settings, VR applications cover a wide range of areas including the neurorehabilitation of patients who are affected by stroke [16,17], Parkinson’s [18] and Alzheimer’s diseases [19], brain injury [20,21], unilateral spatial neglect [22] and pain [23]. VR was also used in psychiatric disorders such as specific phobias [24] and eating disorders [25].

The best results have been achieved for patients suffering from sensorimotor deficits. Given that VR provides the subject with multisensory feedback, the tool may boost neural plasticity within the sensorimotor cortex and may promote functional recovery. Indeed, thanks to the repetitive and task-specific training to acquire knowledge of results and performance, VR increases reinforcement learning (based on rewarding desired behaviors and/or punishing undesired ones), further potentiating both post-brain injury motor and cognitive recovery [26]. Moreover, some VR tools allow the possibility of including an animated avatar inside the hybrid environment. The digital avatar represents a third-person view of the user that appears as a player in the VR environment. The use of such an avatar may boost the plastic changes within the sensory-motor areas that involve the mirror neuron system. It is, indeed, well-known that the observation of an action, even simulated in a virtual place, allows the recruitment of stored motor programs that would promote, in turn, movement execution recovery. This hypothesis was demonstrated by Calabrò et al. [27], who found wide changes in EEG-related α and β oscillation magnitude in the mirror neurons system of stroke patients who underwent motor rehabilitation during a 2D training session guided by a digital avatar.

Despite the increasing enthusiasm for the use of VR for neurorehabilitative applications, the results of systematic reviews varied reporting from insufficient to substantial evidence about the efficacy of VR as superior to traditional treatments; there is a lack of high-quality studies for a clear comprehension of its neurological mechanisms at the basis of this questioned efficacy [21,28]. On the other hand, these results could be affected by the above-mentioned abuse of the term VR. In fact, the reviews often considered data from studies related to nonimmersive 2D screens, which should be simply defined as video-game based therapies or computer-supported therapies, not VR [14]. Moreover, the actual VR applications are characterized by some limitations that should be overcome with the entry of metaverse technology. All the steps necessary for capturing real-time information about the body (movement, the center of pressure, etc.) should be synchronized with the multisensory feedback into the 3D glass (visual and/or acoustic). This process takes time and requires high-quality graphics and huge computational effort to avoid delays between expected and real perception. This phenomenon is called latency of the system and may affect technological VR solutions. Moreover, the subjectively reported distance from a human observer to an object (egocentric distance) is generally underestimated, thus affecting general performance in a VR environment. In the future, new technological devices able to enhance the user’s sense of presence by influencing the accuracy of distance perception will be mandatory [29].

## 4. The Basis for Applying Metaverse-Related Technology in Neurological Rehabilitation 

**Figure 1:**
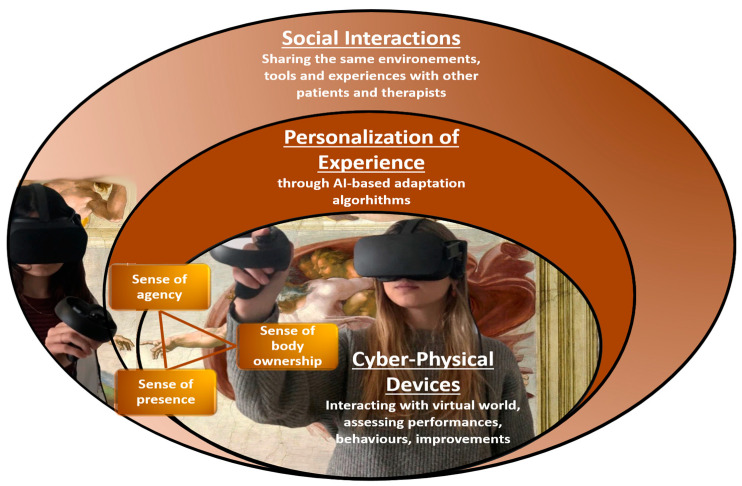
In this illustration, the application of the metaverse in clinical neurorehabilitation is presented as a hierarchical structure whose core is the infrastructure layer, consisting of a new set of wearable sensors and devices that enable the full continuum of physical–digital spaces and contributing to deeper immersive experiences. The personalization layer allows the integration of AI services and other distributed online capabilities, delivering the content of the metaverse therapeutic experience, which needs to be tailored to the specific clinical needs of the patient and adapted as much as possible to his/her individual characteristics. The highest layer allows for social interaction, through web connections, among patients and therapists but also caregivers and familiars, allowing for sharing environments, tools, experiences and data. In this schema, the sense of body ownership is related to the lowest personal layer, the sense of agency links it to the personal experience in the virtual world and the sense of presence refers to own presence but also to the presence of other people in social scenarios.

We believe that the entry of the metaverse into clinical practice will be achieved through notable innovation and the development of new technological devices useful for offering deeper immersive experiences (Figure 1). This new era of innovation could help to overcome the actual limitations reported with VR applications in clinical practice, boosting the efficacy of VR-based treatments with respect to traditional approaches. According to a recent review [30], numerous studies have shown that altering patients’ internal body representations by using the sense of embodiment in a virtual body is a potent tool for modulating some clinical disorders (such as motor, pain or psychiatric disorders). For this reason, we believe that the translation from the metaverse of tools based on the integration of different embodied technologies (e.g., haptic and interoceptive technologies mediated by AI algorithms via digital avatar) will increase the transposition of users’ first-/third-person perspectives, thus enhancing immersive experience. Here, we propose a list of possible neurological disorders where a new generation of advanced VR-based treatment could potentially be applied for overcoming previous technological limitations.

### 4.1. Movement Disorders

We hypothesize that the metaverse-related technology could be applied in neurological disorders where the body schema representation is damaged. Body representation (BR) is a multifaceted concept that is related to the perception, memory and cognition of your body. BR is continuously updated by the different sensory inputs coming from the skin, joint and muscle receptors and flowing to the cerebral cortex through the brain stem, thalamus and cerebellum. At the cerebral cortex level (e.g., in the temporal–parietal cortex) the signals from the visual, vestibular and primary sensory cortices are integrated into an internal body schema. BR consists of both body image (the conscious representation of the body) and body scheme (the dynamic representation of the spatial properties of the body). It is noteworthy that BR is constantly evolving and affects life and interpersonal and social relationships [31]. These premises pave the way for the use of the metaverse in the rehabilitation of both motor and cognitive deficits in patients with different neurological disorders. Indeed, metaverse patients may experience the so-called “embodied cognition”, which has a large spectrum of potentiality in the rehabilitation setting [32]. 

From a neurorehabilitation point of view, metaverse might also give the opportunity to boost motor recovery by exploiting cognitive pathways/resources in a more tailored and sophisticated manner. Motor imagery and action observation are two clear examples of training opportunities that are more powerful when boosted by immersive virtual reality, both for motor recovery after stroke, [33] and improving the motor control of a body powered prosthesis [34]. Moreover, coordination and skill transfer (two important components in sports and in neuromotor rehabilitation) can also be better trained through mental training performed with immersive virtual reality [35,36] 

The metaverse can represent an even greater opportunity in consideration of the senses of agency, body ownership and self-location. The possibility of modifying the first-person vision into a third-person vision and the interaction with other avatars could accelerate processes related to motor learning during rehabilitation by playing even more clearly with the attention based on an internal focus and/or an external focus [37]. The internal focus during rehabilitation is more suitable when the aim is to improve sensory-motor feedback and feedforward components during motor rehabilitation, while the external focus is more suitable when there is a pain maladaptive mechanism that reduces movement intention and action. Therefore, thanks to the metaverse, key aspects of both orthopedic and neurological rehabilitation like attention will be recalled. In fact, to learn motor behavior efficiently, humans rely on interaction of learning and attention, and that might be manipulated in the metaverse [38].

### 4.2. Cognitive Disorders 

One field that could largely benefit from the arrival of the metaverse is cognitive rehabilitation (CR). CR is a way to rehabilitate individuals with brain damage and cognitive problems to compensate for the impairment or recover their normal functioning. CR may be provided in clinical practice in two ways, using either restorative methods or compensative ones. Restorative CR allows the patient to regain their lost cognitive domains by means of specific cognitive training, whereas compensatory CR is based on the use of aids and tools to overcome the deficits. CR is also classified as conventional (when it is based on paper/pencil exercises) or computer-assisted (if innovative devices and software are used) [39].

Generally, individuals immersed in a multisensory stimulation of augmented feedback are more able to obtain both knowledge of results and performance of their training, which are fundamental to reinforcement learning as well as neuroplasticity, and functional recovery [40]. This latter is boosted by the repetitive, intensive and task-oriented training provided by VR. Indeed, VR has been positively applied to different neurological disorders, promoting the recovery of different cognitive functions, including memory, attention, visuospatial cognition, executive processes, and planning [40]. In detail, a recent meta-analysis of 21 studies (1149 participants), VR led to better outcomes (such as MMSE, MoCa, ADL/IADL and FIM scores) than conventional training in patients with different cognitive dysfunction following stroke [41]. This data was corroborated by changes in the event-related potential 300 (P300) amplitude [42]. Concerning multiple sclerosis, it has been demonstrated that the use of VR-exergaming exceeded conventional training for improving cognitive abilities as well as psychosocial status and fatigue [43]. Moreover, VR interventions may be considered beneficial for improving cognitive as well as motor function in individuals with mild cognitive impairment or dementia. This improvement was evident for global cognition, attention/executive function and memory and balance, but VR was not superior in visuospatial ability and gait ability [44]. On the contrary, no conclusive data are available concerning the positive effect of VR in improving motor, cognitive and behavioral function in patients with Parkinson’s disease [45].

Unilateral spatial neglect is another neurological deficit that would benefit from the improvement of VR-related immersive experiences. Indeed, thanks to the possibility to boost embodied cognition using different virtual environments, this may lead to better outcomes in improving neglect and associated symptoms [22,46]. The use of the metaverse, thanks to the realistic experience of “being there” with the therapist, might better work on the attention deficit, not only by potentiating the visual and spatial neglect but also by acting on the body representation, thus potentially improving anosognosia and personal neglect.

### 4.3. Other Neurological Diseases

Another example of a possible condition where the metaverse could be positively applied is frailty. Although this condition is not a disease per se, aging of the brain and other systems may induce behavioral complications that need to be treated for avoiding more severe outcome. In this context, the metaverse would allow for a motivating and multidimensional rehabilitation and socialization. Thanks to the metaverse, older frail individuals could train motor and cognitive problems at the same time, and those individuals with social restriction due to geographical/physical and behavioral/cognitive barriers may benefit from this tool. To this end, the metaverse could be applied during pandemics, like COVID-19, to avoid isolation and treat all kinds of patients in a safer manner. In this case, the adjunctive use of sensors might give clinical staff information regarding the online and offline patients’ performance during training (amount and quality of the movement).

Of particular interest could be the rehabilitation of developmental pathologies such as mild-moderate cerebral palsy. The disease represents the synthesis of the cognitive, sensory, motor and behavioral developmental deficits of a child and would find in the metaverse an opportunity to carry out a true multidimensional rehabilitation, thanks to a well-tested and customized rehabilitation protocol. Many studies have already reported positive results of clinical trials based on digital gaming technologies [47] and virtual reality [48,49] in children with cerebral palsy, as well as studies with the therapist not in person [50]. However, a recent systematic review found that the effect of VR in the rehabilitation of the upper limb of children with CP remains unclear [51]. Although caution is needed for children with cerebral palsy for their risk of epilepsy, this population seems to be an important target for metaverse activities as well as for improving their participation, based on an active inclusion, in schooling and clinical programs based on the new approaches of e-learning, edutainment, gamification [50].

Other clinical conditions in which the metaverse should be used are chronic pain syndromes such as regional complex pain syndrome, where rehabilitation with an external focus can give great benefit, as well as chronic nonspecific cervical and lumbar pain syndrome, where the sharing and awareness of current problems could be learned by the patient through the metaverse (e.g., first lesson of postural cognitive-behavioral re-education such as Back School).

Last but not least, the metaverse could be used for the motor and emotional recovery of injured athletes to better prepare them to return to play by simulating and accustoming them to the stressful conditions of a race. 

Finally, the influence of the metaverse for health care digitalization and artificial intelligence in support of prevention and/or early diagnosis should not be underestimated. In fact, the digital avatar based on biometric data and implemented with other medical information such as blood analysis and imaging tests (Rx, CT, MRI, PET, etc.), could facilitate the aforementioned objectives.

### 4.4. Psychosocial Rehabilitation

The immersion of the patient in the metaverse could be useful not only during the rehabilitation sessions. Patients spent most of their time inactive, alone and confined to their beds during the hospitalization period [52]. Conversely, an enriched environment may favor physical, cognitive and social activities of patients with stroke [53]. The metaverse could provide a virtual enriched environment allowing patients to interact with their relatives at home, with friends and even with other patients in a more stimulating environment. Among the many potential applications, it has been shown as a virtual tour of a museum could improve social inclusion, physical and mental health in older adults [54]. Another interesting application is the possibility of virtually visiting different hospitals before deciding the preferred one [55]. 

A further aspect of the metaverse is its possibility for initial treatment education, during for example a robot-assisted rehabilitation or when learning to use a prosthesis for walking, in order to reduce the time of the learning curve; such education could begin even while the patients are still confined to their beds. Likewise, it is impossible to deny the potential of the metaverse in the education of specialists, particularly in surgery and in medical education at all [56].

## 5. The Metaverse Could Enhance the Translation to the Holistic Neurorehabilitation Approach

In the past, the aim of neurorehabilitation has always been the reduction of harmful effects of motor or cognitive impairments, working on a single deficit using a single device/protocol. In the last ten years, this mono-therapeutic approach has been coupled together with a multidisciplinary approach where the main target is shifted to the patient’s awareness and ability to take motor/cognitive impairments into account in daily living. This holistic approach, which considers the patient’s cognitive, emotional, and psychosocial status, has been regarded as the best rehabilitation approach [57,58]. 

This goal could be achieved through a multidisciplinary strategy where the clinical team participated in the patient’s needs and therapists’ assessment of problems (mobility, self-care ADL, communication, daily occupations, and social interactions). Several studies demonstrated that this kind of inpatient or outpatient rehabilitation programs may reduce disability and bladder dysfunction, and improve participation in neurological patients [59].

Participation is another concept that could be considered in the future application of the metaverse technology in clinical rehabilitation. We here use the term in the context of the World Health organization’s [60] differentiation of “health-related states,” into separate domains: (a) impairment is distinguished from (b) activity limitations (activities) and (c) participation restrictions (participation). Participation is defined as “involvement in life situations,” whereas participation restrictions are defined as “problems an individual may experience in involvement in life situations.” In medical rehabilitation, the degree of participation during neurorehabilitation protocols or at discharge is often neglected. Instead, the new guidelines highlight the need to include participation as one of the main milestones in designing the rehabilitative protocols. Working together improves mood and motivation. This is also called social group work or group therapy, where patients who have similar deficits work together to solve them. This approach is widely employed in the psychiatric domain (i.e., eating disorders, addiction [61]), but in neurological rehabilitation, this is generally neglected. The development of a new technology able to provide a “deep feeling of presence” through a multisensory experience shared by multi users, will revolutionize the employment of medical devices for rehabilitation enhancing the degree of participation in neurological patients, which in turn will promote neural plasticity as well as wellbeing. 

Although social participation and return to work are primary objectives during neuromotor rehabilitation, to date, none of the common rehabilitation strategies is implemented to pursue this objective due to obvious difficulties. The metaverse can represent an optimal environment by simulating scenarios of social and work participation, facilitating the process of integration and the acceptance of one’s different abilities following a trauma of the central nervous system or an amputation, for instance.

## 6. The Pitfalls

The application of unknown technology to neurological disorders hides (obviously) several risks. The first limitation is that the ideal candidates to train with metaverse must have spared cognitive function (i.e., mild or moderate cognitive impairment) as well as the ability to interact with the technology system. Next, the creation of a metaverse-like platform for research purposes is expensive. Third, the validation of AI algorithms used to increase the human-like intelligence of virtual agents in the metaverse platform is a field of study totally unexplored. Moreover, clinicians still have unresolved questions concerning VR diffusion in neurorehabilitation. The threshold of minimal cognitive and perceptual requirements to apply VR for effective sensorimotor rehabilitation is still unclear. Again, different degrees of immersive and augmented reality should have different efficacy that should be clarified in a dedicated systematic review. The AI usually classifies data after training performed on other available data, it could reduce the accuracy of AI for rare disorders. Then, patients could still prefer real social interactions not mediated by virtual technologies. Finally, the great volume of digital personal health data involved in metaverse activities represents huge security and privacy concerns. 

## 7. Conclusions

The application of metaverse technology in clinical neurorehabilitation will surely be a promising field promoting new advances in the clinical translation of VR-based treatments. However, there is an urgent need to perform basic neuroscientific research to solve a large number of pitfalls described above. In other words, we believe that the advent of the metaverse platform could have the merit to stimulate future researchers and clinical applications, but before talking about the beginning of this new era several methodological, technological, and neurophysiological advancements are required.

One of the first applications of the metaverse should be in the robotic neurorehabilitation field. It has widely been demonstrated that robotic-assisted devices boost motor recovery in patients with stroke using end-effector or exoskeleton devices, which are often equipped with immersive 2D experience [62,63]. We believe that motor recovery will be more advanced by using the deeper, persistent, immersive 3D experience mediated by the physical-digital interaction metaverse space with respect to the current VR-based treatments. Again, moving around the immersive virtual experience together with other patients in similar conditions will better stimulate motor imagery and action observational resources.

Telerehabilitation is another field where clinicians and patients would gain advantages from metaverse technologies. Today, the main telehealth systems are equipped with a tablet or devices remotely connected to clinicians. The promise of enabling shared simulated spaces in an immersive clinical setting, where both the therapist and the patient interact by means of an avatar, will stimulate new protocols where the social dimension of the experience could be tailored to the specific clinical needs.

In conclusion, this scoping review is aimed at giving preliminary guidance on how to take advantage of this new technology, considering that raising the degree of immersive experience induces more patients’ participation, interest, and motivation, stimulating additional neural resources.

## Data Availability

Not applicable.

## Abbreviations

VR	Virtual Reality
AI	Artificial Intelligence
AR	Augmented reality
HMD	head-mounted display
